# Inferring circRNA–Disease Associations via Sparse Topological Representation Learning and Dual-View Decoding

**DOI:** 10.3390/ijms27125260

**Published:** 2026-06-10

**Authors:** Chang-Chun Liu, Meng-Meng Wei, Mian-Shuo Lu, Lei Wang

**Affiliations:** 1 Guangxi Key Lab of Human-Machine Interaction and Intelligent Decision, Guangxi Academy of Sciences, Nanning 530007, China; changchunliu@cumt.edu.cn; 2 School of Computer Science and Technology/School of Artificial Intelligence, China University of Mining and Technology, Xuzhou 221116, China; a591286260@gmail.com (M.-M.W.); lumianshuo@cumt.edu.cn (M.-S.L.)

**Keywords:** circRNA–disease association, sparse topological representation learning, dual-view decoding, random walk with restart

## Abstract

Circular RNAs (circRNAs) are key regulators in the onset and progression of complex diseases, offering promise as diagnostic and prognostic biomarkers. However, most putative circRNA–disease associations remain experimentally unverified, largely due to the cost and time demands of wet-lab approaches. To bridge this gap, we present STRCDA (Sparse Topological Representation learning for CircRNA–Disease Associations). The pipeline first constructs fused similarity profiles for circRNAs and diseases by integrating diverse biological attributes. These initial matrices are then refined via random walk with restart to capture local features. Subsequently, a sparse-constrained dual-branch graph autoencoder extracts holistic topological embeddings from the refined local features and the known interaction network. Finally, an XGBoost classifier scores potential circRNA–disease pairs. On the CircR2Disease dataset, STRCDA achieves an AUC of 0.9771 and an AUPR of 0.9826 under five-fold cross-validation. Notably, 18 of the top 20 predicted associations were confirmed by independent experimental evidence, highlighting STRCDA’s efficacy as a robust tool for uncovering circRNA function in disease.

## 1. Introduction

Circular RNAs (circRNAs) constitute a distinctive class of covalently closed, single-stranded transcripts that are typically regarded as non-coding RNAs and are generated through a back-splicing event, in which a downstream 5′ splice donor is ligated to an upstream 3′ splice acceptor to form a circular structure [1,2]. Due to the absence of both a 5′ cap and a 3′ poly(A) tail, circRNAs are inherently resistant to exonucleolytic degradation, exhibiting an intracellular half-life that exceeds that of linear RNA counterparts by more than an order of magnitude; this exceptional stability establishes circRNAs as highly reliable molecular biomarkers [3,4].

Among the various circRNA subtypes, exonic circRNAs represent the most abundant form. Functionally, circRNAs participate in a broad spectrum of regulatory mechanisms: they can modulate immune responses, serve as molecular scaffolds for RNA-binding proteins, function as efficient sponges to sequester microRNAs [5], and, in certain contexts, even translate into biologically active polypeptides through cap-independent initiation pathways [6]. Together, these multifaceted activities position circRNAs as pivotal regulators in disease pathogenesis. In particular, for complex disorders such as cancer, circRNAs have emerged as promising candidates for both diagnostic assessment and therapeutic intervention [7,8,9].

In recent years, a growing number of computational approaches have been developed to overcome the inherent limitations of wet-lab experiments, most notably their protracted experimental cycles and intensive resource consumption. These methods can be broadly categorized into three paradigms: (1) deep learning architectures that autonomously learn hierarchical representations from multimodal omics data, thereby capturing complex, nonlinear patterns without relying on handcrafted features; (2) network propagation-based strategies that systematically exploit biological interaction networks to propagate known association signals and prioritize candidate links; and (3) conventional machine learning models constructed upon manually curated biological features, which often offer enhanced interpretability due to explicit domain knowledge encoding.

As a representative deep learning approach, Lan et al. [10] devised KGANCDA, a knowledge graph attention network that adaptively weighs the importance of neighboring nodes when learning entity embeddings. By aggregating high-order neighborhood features from heterogeneous multi-source associations, it effectively alleviates the data sparsity challenge. Network propagation-based strategies, in contrast, infer associations by exploiting biomolecular networks and their intrinsic topological properties, with core algorithms such as random walk and matrix factorization. For instance, Lu et al. [11] introduced DMFCDA, which integrates both explicit and implicit feedback and employs a multi-layer neural network to model nonlinear relationships between circRNAs and diseases. Traditional machine learning frameworks, on the other hand, rely heavily on feature engineering, typically employing classifiers or regressors and frequently adopting ensemble strategies to enhance generalization. A representative example is RNMFLP, proposed by Peng et al. [12], which couples Robust Non-negative Matrix Factorization with Label Propagation to predict circRNA–disease associations.

Despite the encouraging performance achieved by current approaches, several limitations remain. First, most models adopt a single-scale message-passing scheme during iterative updating, which inherently struggles to concurrently capture fine-grained local topological details and the broader global structural context. Consequently, key biological signals tend to progressively attenuate across successive propagation layers. Second, when trained on sparse datasets, a substantial fraction of hidden neurons contributes negligibly to the final output; yet, owing to the commonly employed activation functions, the neuronal activation rate typically stays above 30%. This redundancy imposes a heavy computational burden during backpropagation and significantly prolongs training convergence, a problem that becomes especially pronounced in biological networks involving long-range dependencies.

**Figure 1:**
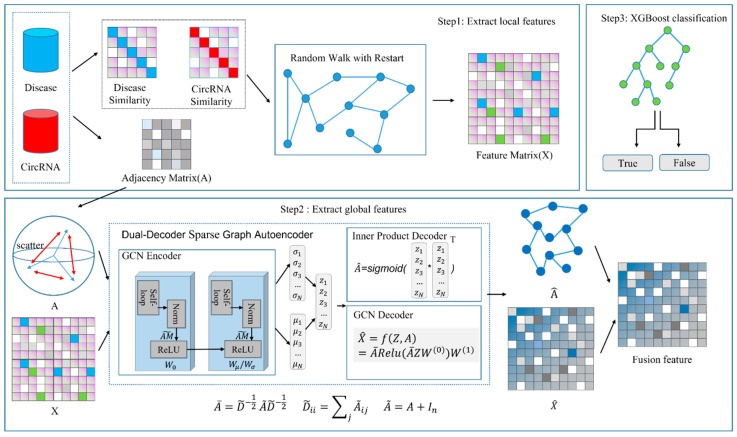
The framework of STRCDA.

To address these challenges, we propose STRCDA, a sparse graph autoencoder framework featuring a dual-decoder architecture. As depicted in Figure 1, the workflow first computes and fuses circRNA-circRNA and disease-disease similarity matrices derived from multi-source biological data. The fused similarity profiles are then refined through a Random Walk with Restart (RWR) procedure to amplify localized structural features. Next, a dual-decoder sparse graph autoencoder jointly learns global contextual representations and local structural embeddings from both the refined similarity features and the known circRNA–disease association graph. Finally, an XGBoost classifier is applied to predict candidate associations.

Central to this framework is the dual-channel decoding design: one decoder reconstructs the adjacency matrix via inner-product operations to capture global topological patterns, while the other employs a graph convolutional decoder to recover the node feature matrix, preserving fine-grained local graph structures. A dynamic weighting mechanism adaptively balances the optimization of these two reconstruction objectives. To improve computational efficiency, we impose L1-norm regularization on the hidden layers of the encoder, which enforces neuron sparsity by retaining only the most crucial connection weights. This sparsification attenuates noise propagation and markedly reduces model complexity.

## 2. Results and Discussion

### 2.1. Evaluation Metrics

In this study, model performance was evaluated using a 5-fold cross-validation strategy and assessed with four quantitative metrics: accuracy (Acc), F1-score (F1), Matthews correlation coefficient (MCC), and the area under the receiver operating characteristic curve (AUC). The first three metrics are defined by

(1) $$Acc=\frac{TP+TN}{TP+TN+FP+FN}$$

(2) $$F1=\frac{2TP}{2TP+FP+FN}$$

(3) $$MCC=\frac{TP\times TN-FP\times FN}{\sqrt{\left(TP+FP)(TP+FN)(TN+FP)(TN+FN\right)}}$$

where TP and TN represent correctly predicted positive and negative samples, while FP and FN denote incorrectly predicted positive and negative samples, respectively. Additionally, we plotted the receiver operating characteristic (ROC) curve and calculated the AUC [13] to provide a more intuitive illustration of the model’s predictive power.

### 2.2. Evaluate Model Performance

**Figure 2:**
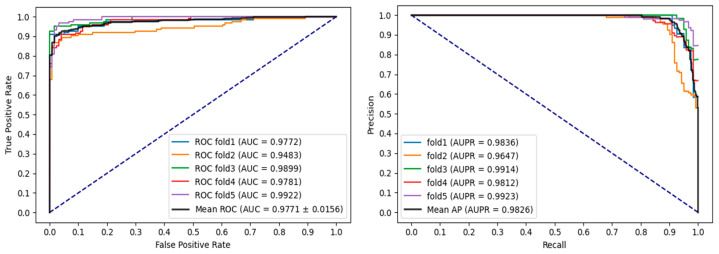
ROC and PR curves of 5-fold CV achieved by STRCDA.

We systematically evaluated the predictive performance of STRCDA for circRNA–disease associations using 5-fold cross-validation on the CircR2Disease benchmark dataset. As summarized in Table 1, the model demonstrated strong and stable classification ability across all folds. Specifically, STRCDA achieved an AUC of 0.9771 ± 0.0156, an AUPR of 0.9826 ± 0.0100, an average F1-score of 92.48% ± 1.44%, an average accuracy of 92.34% ± 1.38%, and an MCC of 84.81% ± 2.84%. The highest individual performance was observed on test set 5, with an AUC of 0.9922, AUPR of 0.9923 and accuracy of 94.63%. The corresponding ROC curves for each fold are presented in Figure 2, with fold-wise AUC values of 0.9772, 0.9483, 0.9899, 0.9781, and 0.9922, respectively. These results collectively indicate that STRCDA delivers consistent, reliable, and highly accurate predictions in identifying circRNA–disease associations, underscoring its potential as a robust tool for circRNA-based biomedical research.

### 2.3. Ablation Study

STRCDA adopts a dual-decoder architecture in which the inner product decoder (Inner decoder) and the GCN decoder function cooperatively. The Inner decoder mainly reconstructs the graph adjacency matrix to capture global topological information, whereas the GCN decoder recovers node features to preserve local structural characteristics. In comparison, VGAE relies on a single decoder that uses variational inference to generate either the graph structure or node features; it lacks a collaborative mechanism for jointly optimizing multidimensional feature spaces, which limits its effectiveness on complex graph data.

To assess the synergy between the Inner and GCN decoders, we performed 5-fold cross-validation and compared their individual performance with that of the combined dual-decoder setup, as reported in Table 2. Among the single-decoder configurations, the GCN decoder exhibited the lowest predictive capability across all evaluation metrics, whereas the Inner decoder achieved substantially higher performance. Importantly, the cooperative combination consistently outperformed both individual decoders on every metric, demonstrating a clear advantage. Specifically, compared with the standalone Inner decoder, the dual-decoder collaboration improved AUC by 0.0380, AUPR by 0.0349 and accuracy by 7.08 percentage points. These results confirm that the dual-decoder design not only provides an overall boost in predictive power but also indicates that the cooperative optimization mechanism effectively strengthens the learning and integration of multidimensional features—a benefit that is especially pronounced on complex graph-structured data.

### 2.4. Comparison of Different Regularization Parameters

To induce sparsity in the autoencoder, we adopted L1 regularization and evaluated its effect by comparing four settings: no sparsity constraint, L1-only, L2-only, and combined L1 + L2 regularization. L1 regularization adds the sum of absolute weight values to the loss, forcing unimportant feature weights toward zero and thereby enabling automatic feature selection and sparsity. L2 regularization adds the sum of squared weights, which prevents overfitting by shrinking large weights but rarely reduces them to zero. The L1 + L2 combination balances sparsity and weight shrinkage, making it more suitable for high-dimensional data with redundant features. Considering both model performance and computational cost, we set the regularization parameter search range from 0.1 to 0.000001.

**Figure 3:**
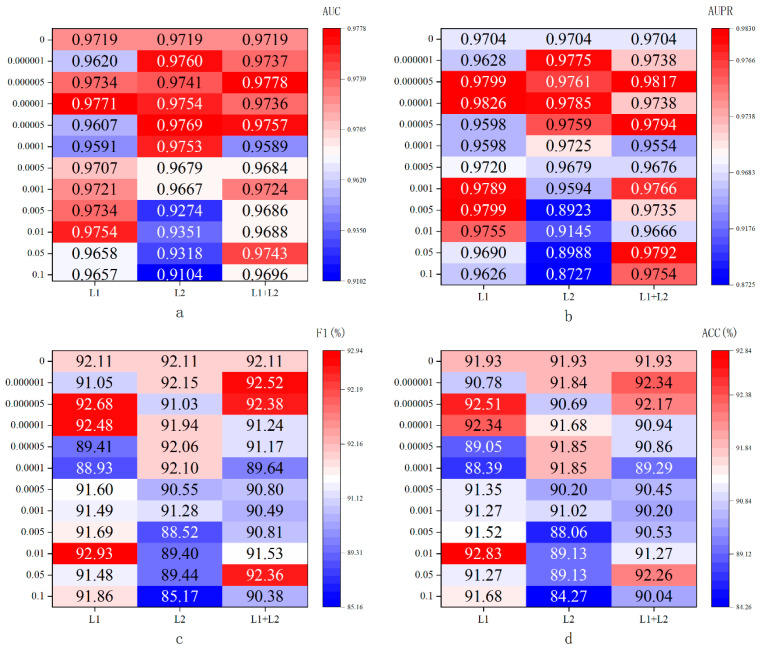
The result of the regularization parameter.

The experimental results shown in Figure 3 indicate that as the regularization parameter decreases, the performance metrics under L1 and L2 generally exhibit a trend of first rising then declining, whereas under L1 + L2, they tend to decline first and then rise. Notably, L1 regularization achieves the best overall performance at a parameter value of 0.00001, yielding an AUC of 0.9771, AUPR of 0.9826, F1-score of 92.48% and accuracy of 92.34%. These findings clearly demonstrate that incorporating L1-induced sparsity into the autoencoder substantially enhances model performance. Furthermore, the results verify the effectiveness of L1 regularization on sparse data, while the L1 + L2 combination is more appropriate for high-dimensional scenarios characterized by redundant features.

### 2.5. Comparison with Different Classifiers

The performance comparison results of the proposed STRCDA with several traditional classifiers (including GBDT, Bagging, AdaBoost, LGBM, and RF) on CDA prediction tasks are shown in Table 3. Among the other classifier methods, random forest (RF) achieves the best performance, with an AUC of 0.9614 and AUPR of 0.9690. Bagging and GBDT rank next, exhibiting comparable performance, while LightGBM (LGBM) lies at an intermediate level. AdaBoost performs significantly worse than the other methods on this task. In contrast, STRCDA consistently outperforms the best RF model across all evaluation metrics: AUC is improved by 0.0157, AUPR by 0.0136, F1-score by 2.21%, accuracy by 2.47%, and MCC by 4.70%. These results clearly demonstrate that the model architecture adopted by STRCDA is more suitable for CDA prediction than conventional classifiers, exhibiting notable advantages in both discriminative ability and robustness.

### 2.6. Performance in Independent Datasets

**Figure 4:**
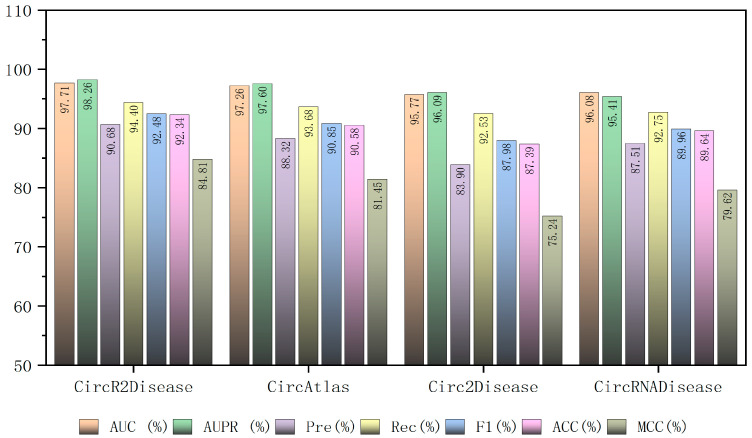
The performance of STRCDA on independent datasets.

To evaluate the generalization capability of STRCDA, we further tested it on three independent CDA datasets: CircAtlas, Circ2Disease, and CircRNADisease. As shown in Figure 4, STRCDA achieved competitive and stable performance across all three datasets, with AUC values of 0.9726, 0.9577, and 0.9608, and AUPR values of 0.9760, 0.9609, and 0.9541, respectively. Notably, the model performed particularly well on the CircAtlas dataset, while maintaining robust results on the other two despite variations in data quality and coverage. These results demonstrate that STRCDA exhibits strong generalization ability, consistently identifying circRNA–disease associations across different independent benchmark datasets without substantial performance degradation.

### 2.7. Comparison with Existing Methods

**Figure 5:**
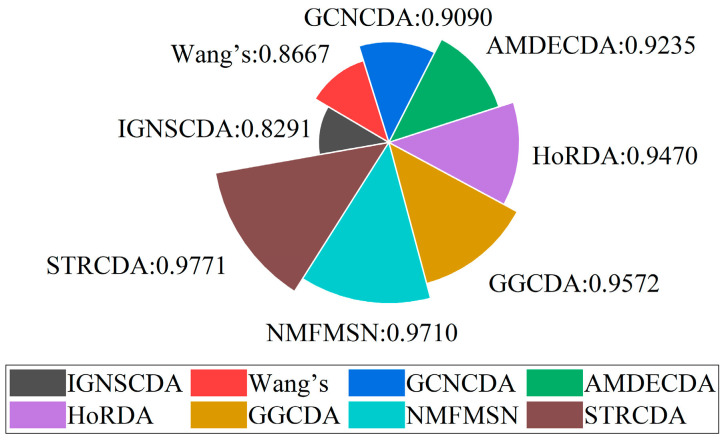
Comparison of AUC obtained by STRCDA and existing methods.

To comprehensively evaluate predictive performance, we benchmarked STRCDA against seven widely used models: HoRDA [14], GCNCDA [15], AMDECDA [16], IGNSCDA [17], NMFMSN [18], GGCDA [19], and Wang’s method [20]. Given that AUC provides a reliable measure of generalization in complex biological settings, it was chosen as the primary comparison metric. The AUC values for STRCDA and the competing approaches are ranked and presented in Figure 5. STRCDA achieved the highest AUC, surpassing the second-best model by 0.0061 and the lowest-ranked model by 0.1480, confirming its superior predictive capability.

### 2.8. Case Study

To assess STRCDA’s ability to discover unknown associations, we made predictions for all pairs in the CircR2Disease dataset, retained the top 20 candidates according to their prediction scores, and confirmed them through literature searches. The results are summarized in Table 4. As shown, 18 of the top 20 predicted pairs were supported by published evidence. This outcome not only underscores STRCDA’s strong efficacy in mining potential circRNA–disease connections, but also demonstrates its capacity to effectively identify unknown associations of biological significance. Consequently, the model supplies a highly reliable candidate set for subsequent wet-lab validation and the discovery of novel disease biomarkers.

## 3. Materials and Methods

### 3.1. Dataset

In this study, raw data were obtained from CircR2Disease and Medical Subject Headings (MeSH) [21], with CircR2Disease serving as the benchmark dataset. This resource is specifically curated for circRNA–disease association research and originally contains 739 connections between 676 circRNAs and 100 diseases. After filtering and cleaning, 561 circRNAs and 607 associations spanning all 100 diseases were retained. The positive set comprises these 607 experimentally validated pairs, while an equal number of unrecorded circRNA–disease pairs were randomly sampled as the negative set, yielding a balanced dataset. Based on these data, a binary adjacency matrix AM was constructed, where $AM\left(i,j\right)=1$ indicates a known association between circRNA $c\left(i\right)$ and disease d(j), and 0 signifies no reported link.

In addition, we used the same strategy to construct independent datasets based on Circ2Disease [22], CircAtlas [23], and CircRNADisease [24] to more comprehensively evaluate the model. Specifically, the CircAtlas dataset contains 708 circRNAs, 117 diseases, and 775 experimentally validated associations; the Circ2Disease dataset contains 234 circRNAs, 60 diseases, and 254 experimentally validated associations; and the CircRNADisease dataset contains 286 circRNAs, 48 diseases, and 304 experimentally validated associations. For each dataset, equal numbers of unrecorded circRNA–disease pairs were randomly selected as negative samples to construct balanced datasets for model evaluation.

### 3.2. Construction of Similarity

It is well-established that diseases with similar pathological features tend to involve functionally analogous circRNAs [25]. Therefore, quantifying the similarity between circRNAs and diseases can provide valuable clues for predicting their associations. Accordingly, in this study, we constructed four types of similarity measures: circRNA functional similarity, circRNA Gaussian interaction profile kernel (GIPK) similarity, disease semantic similarity, and disease GIPK similarity.

*(1) CircRNA functional similarity:* Given that circRNAs associated with semantically similar diseases often exhibit shared functional characteristics, we measure circRNA functional similarity as follows. For circRNAs c(i) and c(j), let D(i) and $D\left(j\right)$ denote the sets of diseases related to c(i) and c(j), respectively. First, the similarity between a disease d and a disease set D is defined as the maximum semantic similarity within the set:

(4) $$S(d\left(i\right),D\left(j\right))={max}_{1\leq k\leq \left|D\left(j\right)\right|}(DSS(d\left(i\right),d(k)))$$

Then, the functional similarity $CFS\left(c\left(i\right),c\left(j\right)\right)$ is obtained by averaging the cross-set similarities of all diseases in D(i) against D(j) and vice versa:

(5) $$CFS\left(c\left(i\right),c\left(j\right)\right)=\frac{\sum_{1\leq i\leq \left|D(i)\right|}S(d\left(i\right),D(j))+\sum_{1\leq j\leq \left|D(j)\right|}S(d\left(j\right),D(i))}{\left|D(i)\right|+\left|D(j)\right|}$$

*(2) CircRNA GIPK similarity:* The computation of circRNA GIPK similarity adopts the same framework used for disease GIPK similarity [26,27]. Specifically, the GIPK similarity between circRNA c(i) and c(j), denoted as CGS(c(i),c(j)), is given by

(6) $$CGS(c(i),c(j))=exp(-\theta_{c}{\left\|V(c(i))-V(c(j))\right\|}^{2})$$

(7) $$\theta_{c}=\frac{1}{m}\sum_{i=1}^{m}{\left||V(c(i))|\right|}^{2}$$

where m denotes the total number of circRNAs in the adjacency matrix AM, and $\theta_{c}$ acts as a regularization parameter that controls the kernel bandwidth.

*(3) Disease semantic similarity:* We establish a quantitative model for disease semantic similarity by leveraging the MeSH hierarchical structure, which incorporates main headings, subheadings, and synonyms [28,29]. From the MeSH data, a Directed Acyclic Graph (DAG) is constructed to represent disease relationships. For a given disease d, its DAG is represented as ${DAG}_{d}=(d,N_{d},\;E_{d})$, where ${\;N}_{d}$ is the set of diseases related to d, and $E_{d}$ is the set of edges connecting these nodes. Let disease a be an element of this DAG; its semantic contribution to d, denoted $D_{d}\left(a\right)$, is defined as

(8) Dda=1max(μ·Dd(a′)|a′∈children of a)             if a=dif a≠d

Here, μ is the semantic contribution factor assigned to the edges in $E_{d}$ that link disease a with its child diseases $a^{\prime }$. The overall semantic content of disease d is then computed as

(9) $${DS}_{d}\left(d\right)=\sum_{a\in N_{d}}D_{d}(a)$$

For two diseases $d\left(i\right)$ and $d\left(j\right)$, their semantic similarity $DSS(d\left(i\right),d(j))$ is calculated by

(10) $$DSS(d\left(i\right),d(j))=\frac{\sum_{a\in N_{d(i)}\cap N_{d(j)}}{(D}_{d\left(i\right)}(a)+D_{d\left(j\right)}(a))}{{DS}_{d}\left(d\left(i\right)\right)+{DS}_{d}(d(j))}$$

*(4) Disease GIPK similarity:* We built a disease similarity model from the circRNA–disease adjacency matrix AM using the GIPK. For each disease d(i), a binary interaction profile vector V(d(i)) was derived, with each entry set to 1 if an association with the corresponding circRNA is recorded, and 0 otherwise. The GIPK similarity DGS(d(i),d(j)) between two diseases d(i) and d(j) is then defined as follows:

(11) $$DGS(d(i),d(j))=exp(-\theta_{d}{\left\|V\left(d\left(i\right)\right)-V\left(d\left(j\right)\right)\right\|}^{2})$$

(12) $$\theta_{d}=\frac{1}{m}\sum_{i=1}^{m}{\left\|V(d(i))\right\|}^{2}$$

where m is the total number of diseases in AM, and $\theta_{d}$ acts as a regularization parameter that controls the kernel bandwidth.

### 3.3. Multi-Source Similarity Fusion

To integrate multi-source information more effectively, we adopt a multi-similarity matrix fusion strategy that combines circRNA and disease similarity data, enabling feature complementation [30,31]. This fusion design captures the distinctive characteristics of different data sources while reducing the limitations inherent in relying on a single type of feature.

For diseases, the fused similarity $DS\left(d\left(i\right),d\left(j\right)\right)$ is defined by prioritizing semantic similarity: if $DSS(d\left(i\right),d(j))$ is nonzero, it is used directly; otherwise, the GIPK similarity DGS(d(i),d(j)) is employed instead. This rule is expressed as

(13) DSdi,dj=DSS(di,d(j))DGS(d(i),d(j))    if DSS(d(i),d(j))≠0otherwise

CircRNA similarities are fused in an analogous manner. The combined similarity $CS\left(c\left(i\right),c(j)\right)$ takes the value of functional similarity $CFS(c\left(i\right),c(j))$ whenever it exists (nonzero), and falls back to the GIPK similarity CGS(c(i),c(j)) otherwise, as formalized below:

(14) CSci,c(j)=CFS(ci,c(j))CGS(c(i),c(j))    if CFS(c(i),c(j))≠0otherwise

### 3.4. Local Feature Extraction via RWR

Rooted in Markov chain diffusion, the Random Walk with Restart (RWR) algorithm quantifies node relevance through iterative transition probability updates. By incorporating a restart parameter, the walker can dynamically balance local neighborhood exploration with the preservation of global structure. The restart parameter tunes this trade-off, while the teleportation mechanism attenuates noise, enabling robust feature extraction from sparse networks.

In our framework, we realize a topology fusion strategy by applying RWR on the similarity matrix. The resulting matrix is then concatenated column-wise with the adjacency matrix to construct a local feature representation. This hybrid integration scheme effectively retains both local neighborhood structures and global semantic relationships embedded in the graph topology.

### 3.5. Dual-Decoder Sparse Graph Autoencoder

The Variational Graph Autoencoder (VGAE) integrates graph convolutional networks with variational inference to derive latent representations from graph-structured data, supporting tasks such as node embedding, link prediction, and graph reconstruction. Operating on a graph defined by nodes and an adjacency matrix, VGAE employs neighborhood aggregation via graph convolution layers to encode node features into distribution parameters. These parameters specify Gaussian distributions from which latent vectors are sampled, enabling gradient-based optimization, while a decoder reconstructs edge probabilities through inner products between node embeddings. This architecture offers scalability across diverse graphs and maintains probabilistic interpretability. However, the conventional VGAE relies on a single-decoder design to generate either graph topology or node features, lacking collaborative optimization across multidimensional feature spaces.

To address this limitation, we propose STRCDA, which introduces a dual-decoder cooperative framework that jointly reconstructs both the node feature matrix and the graph adjacency matrix. A two-branch decoding mechanism concurrently optimizes feature-space representations and topological relationships, enhancing the integrity of graph data embeddings while ensuring structural consistency under sparsity constraints. By decoupling node feature recovery from structure optimization and coupling their backpropagation, the model improves feature interpretability and noise robustness. Moreover, we incorporate L1-regularized sparsity by adding a sparse penalty term to the reconstruction loss, which restricts the simultaneous activation of all hidden units during decoding. This effectively balances enhanced noise resistance with improved computational efficiency.

Here, we feed the feature matrix X and the adjacency matrix A into the STRCDA encoder. The encoder integrates node attributes with structural information through two graph convolutional layers. The first layer is defined as follows:

(15) $$\bar{X}=GCN\left(X,A\right)=ReLU(\tilde{A}XW_{0})$$

(16) $$\tilde{A}=D^{-\frac{1}{2}}(A+I)D^{-\frac{1}{2}}$$

where $W_{0}$ denotes the trainable weight matrix of the first GCN layer, and D is the degree matrix of the adjacency matrix AM.

The latent representation z is then computed via the reparameterization trick. First, the mean vector μ and the logarithm of the standard deviation σ are produced by linear transformations of the normalized and propagated features:

(17) $$\mu =\tilde{A}\bar{X}W_{\mu }$$

(18) $$log\sigma =\tilde{A}\bar{X}W_{\sigma }$$

Subsequently, z is sampled as

(19) z=μ+σ∗ε

where ε is drawn from a standard normal distribution, ε~N(0,1).

During the decoding stage, we employ an inner product decoder to reconstruct the adjacency matrix and a GCN decoder to recover the node features, as defined below:

(20) $$\hat{A}=Sigmoid(zz^{T})$$

(21) $$\hat{X}=\tilde{A}ReLU(\tilde{A}XW_{0})W^{\left(1\right)}$$

To promote sparsity in the model, we augment the loss function with an L1 regularization term alongside the reconstruction error and Kullback–Leibler (KL) divergence. L1 regularization penalizes the sum of the absolute weight values in the objective, pushing the weights toward zero and encouraging sparse representations. In deep learning and machine learning, L1 regularization is commonly used for feature selection and model simplification, as it suppresses irrelevant features and parameters, thereby enhancing generalization and reducing overfitting. In our model, the L1 penalty eliminates unnecessary complexity, improving training efficiency and making the final model more interpretable. Reconstruction quality is measured using weighted cross-entropy, while the KL divergence regularizes the latent space structure. Together, these components enable the model to learn the graph structure effectively while preserving strong generalization capability. The loss function is defined as

(22) $$L=E_{q\left(z|X,A\right)}\left[\log_{\rho }\left(A|Z\right)\right]-KL\left[q\left(Z|X,A\right)||p(Z)\right]+\varphi \sum_{i=1}^{n}\left|W_{i}\right|$$

where φ is a regularization parameter that controls the contribution of the L1 penalty term.

## 4. Conclusions

CircRNAs have the potential to serve as biomarkers for a wide range of diseases. In this work, we present STRCDA, a novel approach built around a dual-decoder design. One branch, an inner product decoder, reconstructs the adjacency matrix to capture global topological patterns, while the other, a graph convolutional decoder, recovers the node feature matrix to preserve local structural detail. By jointly optimizing both the structural and feature spaces of the circRNA–disease network, this dual-decoder strategy yields more comprehensive and faithful reconstructions. The model further incorporates L1 regularization directly into the loss function as a sparsity-inducing penalty. This encourages sparse weight solutions, curtailing the number of actively engaged neurons. As a result, model complexity and overfitting are reduced, the most relevant features and pathways for circRNA–disease associations are highlighted for better interpretability, and computational efficiency is improved by eliminating redundant neuronal activations. Collectively, these design choices substantially boost overall efficiency and predictive accuracy, establishing STRCDA as a powerful tool for CDA prediction.

Despite its strong performance, STRCDA has several limitations. Its dependence on known association data limits predictive accuracy for rare diseases, where such information is scarce. Moreover, the dual-decoder architecture introduces higher computational overhead when processing feature matrices, leading to markedly longer training times as the dataset size grows. We anticipate that ongoing refinements to the model will further mitigate these shortcomings and enhance its capabilities.

## Figures and Tables

**Table 1:** Result of 5-fold CV achieved by STRCDA.

Test Set	AUC	AUPR	F1 (%)	Acc (%)	MCC (%)
1	0.9772	0.9836	92.8	92.59	85.29
2	0.9483	0.9647	91.29	91.36	82.74
3	0.9899	0.9914	92.8	92.59	85.37
4	0.9781	0.9812	90.69	90.53	81.14
5	0.9922	0.9923	94.82	94.63	89.5
Average	0.9771 ± 0.0156	0.9826 ± 0.0100	92.48 ± 1.44	92.34 ± 1.38	84.81 ± 2.84

**Table 2:** Result of ablation study.

Decoder	AUC	AUPR	F1 (%)	Acc (%)	MCC (%)
Inner	0.9391 ± 0.0118	0.9477 ± 0.0086	86.09 ± 1.45	85.26 ± 1.71	71.17 ± 3.24
GCN	0.9273 ± 0.0132	0.9287 ± 0.0087	84.23 ± 2.01	83.36 ± 2.04	67.22 ± 4.26
Inner + GCN	0.9771 ± 0.0156	0.9826 ± 0.0100	92.48 ± 1.44	92.34 ± 1.38	84.81 ± 2.84

**Table 3:** Comparison results of different classifier models.

Classifier	AUC	AUPR	F1 (%)	Acc (%)	MCC (%)
Bagging	0.9569 ± 0.0191	0.9645 ± 0.0145	89.97 ± 2.37	89.54 ± 2.62	79.44 ± 5.04
AdaBoost	0.7609 ± 0.0343	0.7377 ± 0.0383	67.70 ± 2.12	68.21 ± 2.48	36.46 ± 5.01
LGBM	0.9259 ± 0.0076	0.9160 ± 0.0063	85.92 ± 0.85	85.50 ± 0.88	71.25 ± 1.76
RF	0.9614 ± 0.0172	0.9690 ± 0.0125	90.27 ± 1.44	89.87 ± 1.45	80.11 ± 2.98
GBDT	0.9425 ± 0.0179	0.9225 ± 0.0292	89.13 ± 1.61	88.63 ± 1.65	77.61 ± 3.36
STRCDA	0.9771 ± 0.0156	0.9826 ± 0.0100	92.48 ± 1.44	92.34 ± 1.38	84.81 ± 2.84

**Table 4:** The top 20 circRNA–disease associations predicted by STRCDA.

Rank	circRNA	Disease	Evidence (PMID)
1	hsa_circ_0001946	Osteosarcoma	30425578
2	hsa_circ_0001946	Breast cancer	41010991
3	hsa_circ_0001946	Glioma	31599076
4	hsa_circ_0001445	Gastric cancer	30956729
5	hsa_circ_001763	Gastric cancer	33060778
6	hsa_circ_0023404	Endometrial cancer	36352482
7	hsa_circ_0001445	Glioma	34198978
8	circSMARCA5	Gastric cancer	35515212
9	circBCL11B	Oral squamous cell carcinoma	34288804
10	circSMARCA5	Glioblastoma	36430152
11	hsa_circ_0000284	Glioma	33194066
12	circFAT1	Lung adenocarcinoma	35844799
13	hsa_circ_0000520	Gastric cancer	29103021
14	hsa_circ_0007534	Gastric cancer	32419229
15	hsa_circ_0047905	Glioma	N/A
16	hsa-circRNA7690-15	Glioma	N/A
17	hsa_circ_0006988	Hepatocellular carcinoma	36594050
18	circ-Foxo3	Gastric cancer	37702694
19	Cir-ITCH	Gastric cancer	33060778
20	circRHOBTB3	Pancreatic ductal adenocarcinoma	34416910

## Data Availability

Data Availability Statement: The datasets and source code used in this study are publicly available at https://github.com/591286260/STRCDA (accessed on 7 June 2026). Further inquiries can be directed to the corresponding author.
